# ELF1‐mediated LUCAT1 promotes choroidal melanoma by modulating RBX1 expression

**DOI:** 10.1002/cam4.2859

**Published:** 2020-01-22

**Authors:** Lina Wang, Dongrun Tang, Tong Wu, Fengyuan Sun

**Affiliations:** ^1^ Tianjin Medical University Eye Hospital Tianjin China; ^2^ Tianjin First Central Hospital Tianjin China

**Keywords:** choroidal melanoma, ELF1, LUCAT1, miR‐514a/b‐3p, RBX1

## Abstract

Long noncoding RNAs (lncRNAs) are essential regulators of gene expression and biological behaviors. However, the contribution of lncRNA LUCAT1 to choroidal melanoma (CM) remains unexplored. Here, we examined the expression of LUCAT1 in CM cells by qRT‐PCR and investigated its biological effects by cell counting kit‐8, EdU, TUNEL, transwell assays, and Western blot. Bioinformatics tools were applied to find RNA candidates for further study. Moreover, mechanistic experiments including RNA immunoprecipitation assay, pull‐down assay, and luciferase reporter assay confirmed the relation or interaction among the indicated molecules. Here, we reported ELF1 as the transcription activator of LUCAT1. Functionally, elevated expression of LUCAT1 positively regulated CM cell proliferation, metastasis, and epithelial‐mesenchymal transition process. In addition, we verified the competing endogenous RNA (ceRNA) hypothesis of LUCAT1 and confirmed LUCAT1 modulates CM progression by modulating miR‐514a/b‐3p/RBX1 axis. Meanwhile, miR‐514a/b‐3p was suggested to repress CM progression, whereas RBX1 was unmasked to aggravate CM development. Of note, RBX1 overexpression rescued the inhibitory effect of LUCAT1 silence on the biological processes of CM cells. Altogether, this study unveiled the modulation axis ELF1/LUCAT1/miR‐514a/b‐3p/RBX1 and evidenced LUCAT1 as a promoter in CM for the first time, providing a novel insight into future treatment of CM.

## Graphical abstract

LUCAT1 modulates miR‐514a/b‐3p expression by serving as a sponge in CM. RBX1 is the downstream target gene of miR‐514a/b‐3p in CM.

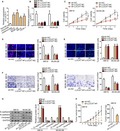

## 1 INTRODUCTION

Choroid melanoma (CM) is the most common intraocular malignancy occurring among the adults. The overall CM incidence of occurrence is around 20 per million cases each year.^1^ Mortality rate for patients with CM is around 50% due to its latency and metastatic potential, which usually involves the liver.^2^ The median survival for choroidal malignant melanoma, the malignant type of CM, is 4‐5 months. Such dismal prognosis is resulted from various clinical factors including tumor size.^3^ Therefore, exploring the recognition of effective CM‐related molecules and the underlying mechanisms for improvement of this disease is of great significance.

Long noncoding RNAs (lncRNAs) are a subgroup under noncoding RNAs with transcripts exceeding 200 nucleotides in length and limited protein‐coding capacity.^4^, ^5^, ^6^ Development of application of next‐generation sequencing has identified thousands of lncRNAs and revealed that the aberrantly expressed lncRNAs are correlated with different cancer types. Notably, these lncRNAs are crucial regulators in gene regulation and accordingly modulate various aspects of cellular homeostasis such as proliferation, migration, invasion, and epithelial‐mesenchymal transition (EMT) process.^7^, ^8^ For example, lncRNA LUCAT1 promotes esophageal squamous cell carcinoma metastasis.^9^ LUCAT1 drives glioma progression by promoting cell viability and invasion.^10^ As to CM, previous study has reported that lncRNA FOXCUT coordinates with miR‐296‐3p to target MMP‐2/MMP‐9 and exerts influence on choroidal malignant melanoma.^3^ Although lncRNAs have been demonstrated playing an essential role in CM, the precise effects and mechanism of LUCAT1 in CM remain largely unknown.

In this study, we investigated the expression of LUCAT1 in CM cells and detected the upregulated expression in cancerous cells compared to that in normal choroidal melanocytes. To sum up, we identified LUCAT1 as an unfavorable biomarker for diagnosis of CM.

## 2 MATERIALS AND METHODS

### 2.1 Cell lines

Normal choroidal melanocytes and CM cell lines (C918, MUM‐2B, M619, OCM‐1) were maintained in Dulbecco's Modified Essential Medium (DMEM; Invitrogen) at 37°C in 5% CO_2_, procured from Shanghai Institute of Cell Biology. Streptomycin (100 μg/mL) and penicillin G (100 U/mL), as well as 10% fetal bovine serum (FBS; Thermo Fisher Scientific), were used as medium supplements.

### 2.2 Quantitative real‐time PCR

M619 and MUM‐2B were cultured in TRIzol reagent (Invitrogen) for extracting total RNAs following the established protocol. The cDNA was then synthesized by TaKaRa Reverse Transcription Kit (TaKaRa) for qPCR procedure with the Power SYBR Green (TaKaRa) on Step‐One Plus System (Applied Biosystems). All results were calculated by the comparative 2^−ΔΔCt^ method and normalized to U6 or GAPDH. Analyses were carried out in triplicate and repeated three times.

### 2.3 Transfection plasmids

M619 and MUM‐2B cells were cultured to about 70% confluence in the 6‐well plates for 48 hours of transfection using Lipofectamine2000 (Invitrogen). The short hairpin RNAs (shRNAs) targeting LUCAT1 (sh‐LUCAT1#1/2/3), ELF1 (sh‐ELF1), RBX1 (sh‐RBX1#1/2/3), and control shRNAs (sh‐NC) were acquired from RiboBio for silencing gene. The miR‐514a/b‐3p mimics and control (miR‐NC), pcDNA3.1/RBX1 (OE/RBX1), pcDNA3.1/ELF1 (OE/ELF1), and empty pcDNA3.1 vector were obtained from Genepharma for overexpressing gene.

### 2.4 Cell counting kit‐8

Choroidal melanoma cells were incubated with 10 μL of cell counting kit‐8 (CCK‐8) reagent (Beyotime) for 2 hours at 37°C in the 96‐well culture plates (5 × 10^3^/well). Cell viability was determined by evaluating the optical density value at 450 nm with microplate reader. The experiments were performed in triplicate for three repeats.

### 2.5 EdU incorporation assay

Transfected CM cells were treated with EdU medium diluent (Ribobio) for 3 hours, then with 4% paraformaldehyde and 0.5% Troxin X‐100. After staining with Apollo^®^ 488 fluorescent at 37°C for 30 minutes, DAPI (Beyotime) was utilized for nuclear staining, followed by visualization under fluorescent microscope (Leica). Three repeated EdU assays were conducted indistinguishably to assess the proliferation of different cells.

### 2.6 TUNEL staining assay

The fixed CM cells were prepared in 0.2% Triton X‐100 and treated with dUTP‐end labeling (Clontech), following DAPI staining. The stained cells were analyzed using fluorescent microscope, and finally, results were obtained from three repetitions of such staining.

### 2.7 Transwell assays

Transwell inserts with or not Matrigel (BD Biosciences) coating were bought from Corning Co for cell invasion or migration assay. CM cells were placed into the upper chamber in 24‐well plates, while the lower chamber was filled with medium with 20% FBS. After 48 hours of incubation, the invading or migrating CM cells were subjected to 4% paraformaldehyde and 0.1% crystal violet prior to observation with fluorescence microscope. Assays were repeated three times.

### 2.8 Western blotting

Cellular protein samples in RIPA buffer were separated via electrophoresis on 10% SDS‐PAGE and loaded onto PVDF membranes (Millipore), following treatment with 5% skimmed milk for 2 hours. The primary antibodies against E‐cadherin, N‐cadherin, Vimentin, ELF1, and GAPDH were all obtained from Abcam, along with the HRP‐labeled secondary antibodies. After three washes in phosphate buffer saline, band density was monitored by enhanced chemiluminescence reagent (Santa Cruz Biotechnology). Each sample had three duplicates each time and the experiment was performed for three times.

### 2.9 Xenograft assay

Six‐week‐old of male nude mice, purchased from the National Laboratory Animal Center, were maintained in SPF‐grade lab, with the approval of the Animal Research Ethics Committee of Tianjin First Central Hospital. Xenograft assay was carried out by subcutaneous injection of 5 × 10^6^ transfected M619 cells into nude mice. After 28 days of inoculation, mice were killed before tumors were carefully excised for weighing. Tumor volume was recorded as 0.5 × length × width^2^.aaa

### 2.10 Chromatin immunoprecipitation

M619 and MUM‐2B cells were subjected to formaldehyde, then the DNA‐protein cross‐links were broken into 200‐ to 500‐bp fragments. Immunoprecipitation was conducted with 2 μg of anti‐ELF1 and 2 μg of corresponding control anti‐IgG all night at 4°C, followed by adding the magnetic beads for 2 hours. At last, the precipitated chromatin was assayed by quantitative real‐time PCR (qRT‐PCR) and electrophoresis. Above steps were repeatedly carried out for three times.

### 2.11 Dual‐luciferase reporter assays

To conduct LUCAT1 promoter luciferase analysis, M619 and MUM‐2B cells in 24‐well plates were co‐cultured with pGL3‐Basis vector containing LUCAT1 promoter, pRL‐TK‐Renilla (Promega) sh‐ELF1, and sh‐NC for 48 hours. Besides, the wild‐type (Wt) or mutant (Mut) miR‐514a/b‐3p binding sites to LUCAT1 sequence were formed for cloning to pmirGLO Dual‐Luciferase Expression Vector (Promega), named as LUCAT1 Wt/Mut. The reporter vectors RBX1 Wt/Mut were generated as above described. After cotransfection, Dual‐Luciferase Reporter Assay System (Promega) was employed for examining luciferase activity. The experiment was repeated three times.

### 2.12 Subcellular fractionation assay

Subcellular fractionation assay was conducted in CM cells using Invitrogen PARIS™ Kit as per the guidelines. Lysed cells in cell fraction buffer were treated with centrifugation to collect the supernatant. The remaining lysates were incubated with cell disruption buffer to obtain cell nuclei. Levels of LUCAT1, GAPDH, and U6 were analyzed by qRT‐PCR. Experiments were repeated from three times.aaa

### 2.13 RNA immunoprecipitation

RNA immunoprecipitation (RIP) assay was performed in CM cells in line with the protocol of Magna RIP™ RNA‐Binding Protein Immunoprecipitation Kit (Millipore). 1 × 10^7^ cells collected from RIP lysis buffer were immunoprecipitated with anti‐Ago2 or anti‐IgG antibody (Millipore). The recovered RNA by magnetic beads was detected by qRT‐PCR. Data collected from three repeats of this assay were finally analyzed.

### 2.14 RNA pull‐down assay

Pierce Magnetic RNA‐Protein Pull‐Down Kit was bought from Thermo Fisher Scientific for RNA pull‐down assay. The cellular protein samples of CM cells were cultivated with the biotinylated RNAs (Bio‐miR‐514b‐3p or Bio‐NC) in the presence of magnetic beads at 4°C for 1 hour. The complex of RNA protein was measured by qRT‐PCR. This experiment was conducted for three times.

### 2.15 Statistical analysis

Each assay contained at least three biological repeats. Results were assayed with ANOVA (one‐way or two‐way) or unpaired *t* test utilizing PRISM 7 (GraphPad), with *P* < .05 as cutoff level. All data were expressed as the mean ± SD.

## 3 RESULTS

### 3.1 Elevated LUCAT1 expression is correlated with the malignant phenotype of choroidal melanoma

**Figure 1:**
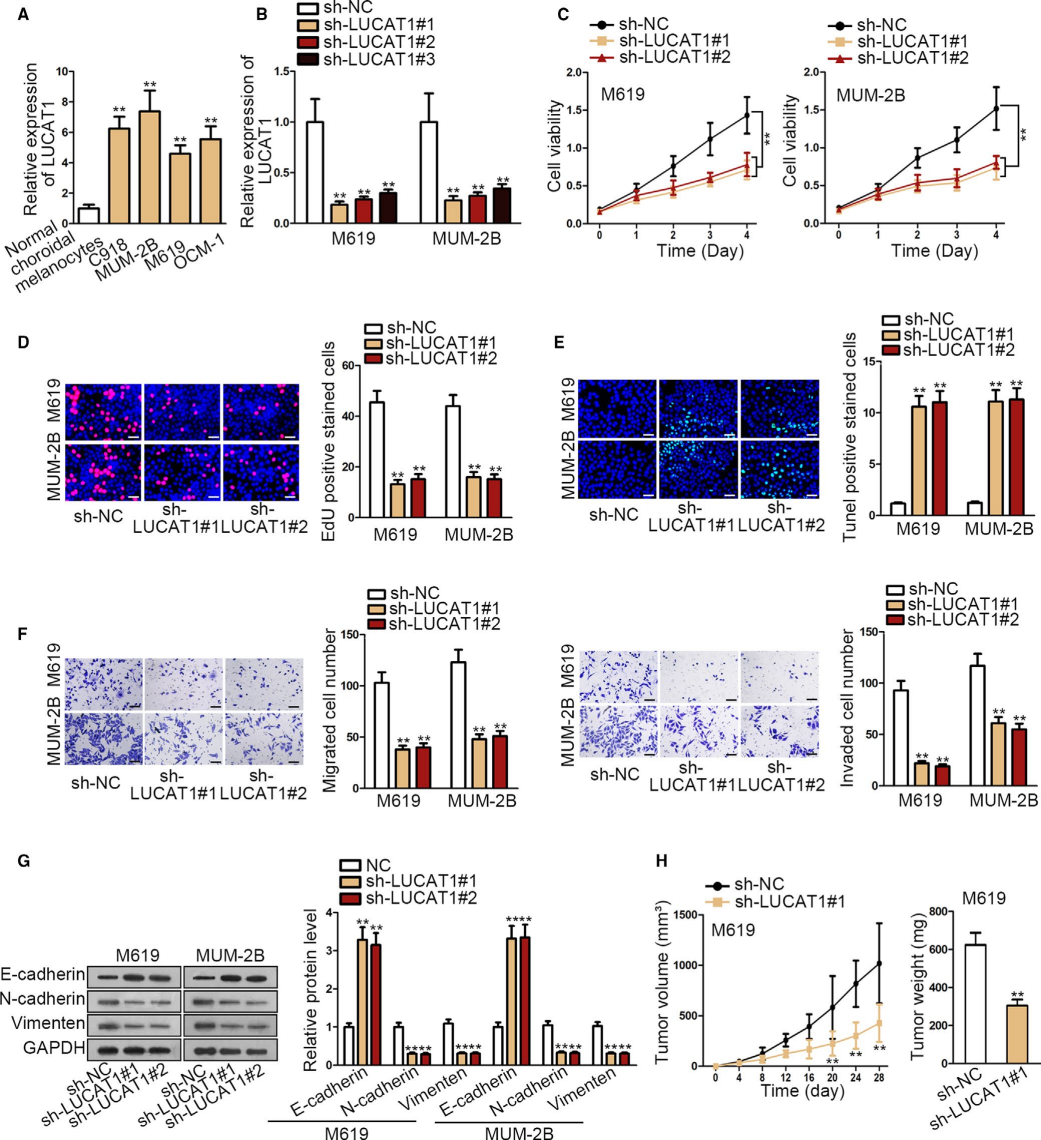
Elevated LUCAT1 expression is correlated with the malignant phenotype of choroidal melanoma. A, The expression of LUCAT1 in choroidal melanoma cells detected by qRT‐PCR. B, LUCAT1 interference efficiency analysis. C‐E, Cell viability, proliferation, and apoptosis were detected by after silencing LUCAT1 via CCK‐8, EdU (scale bar = 200 μm), and TUNEL (scale bar = 200 μm). F, Cell migration and invasion ability were analyzed by transwell assays (scale bar = 200 μm). G, Western blot assay detected EMT process. H, A xenograft model was established to examine the effects of silencing LUCAT1 in vivo. **P < .01

LUCAT1 is widely considered as an oncogene in various cancers. However, the contribution of LUCAT1 to CM remains largely unknown. To start our study, we detected the expression of LUCAT1 in CM cells, finding LUCAT1 was prominently elevated in cancerous cells compared to normal cells (Figure 1A). To further confirm the impact of LUCAT1 on CM, functional experiments were carried out next. After examining the interfering efficacy of shRNAs targeting LUCAT1 (Figure 1B), we discovered that silencing LUCAT1 attenuated cell viability and proliferation (Figure 1C,D). By performing T**UNEL** assay, we observed an obvious increase in cell apoptosis in face of LUCAT1 inhibition (Figure 1E). Moreover, migrated and invaded cell number was prominently decreased after knockdown of LUCAT1 according to the results of transwell assays (Figure 1F). Moreover, we also revealed that silencing LUCAT1 slowed down EMT process (Figure 1G). In addition, the in vivo experiments further demonstrated that depletion of LUCAT1 hindered tumor growth in vivo, evidenced by resultantly reduced tumor volume and weight (Figure 1H). In conclusion, LUCAT1 is an oncogenic regulator in CM.

### 3.2 ELF1 is the transcription activator of LUCAT1

**Figure 2:**
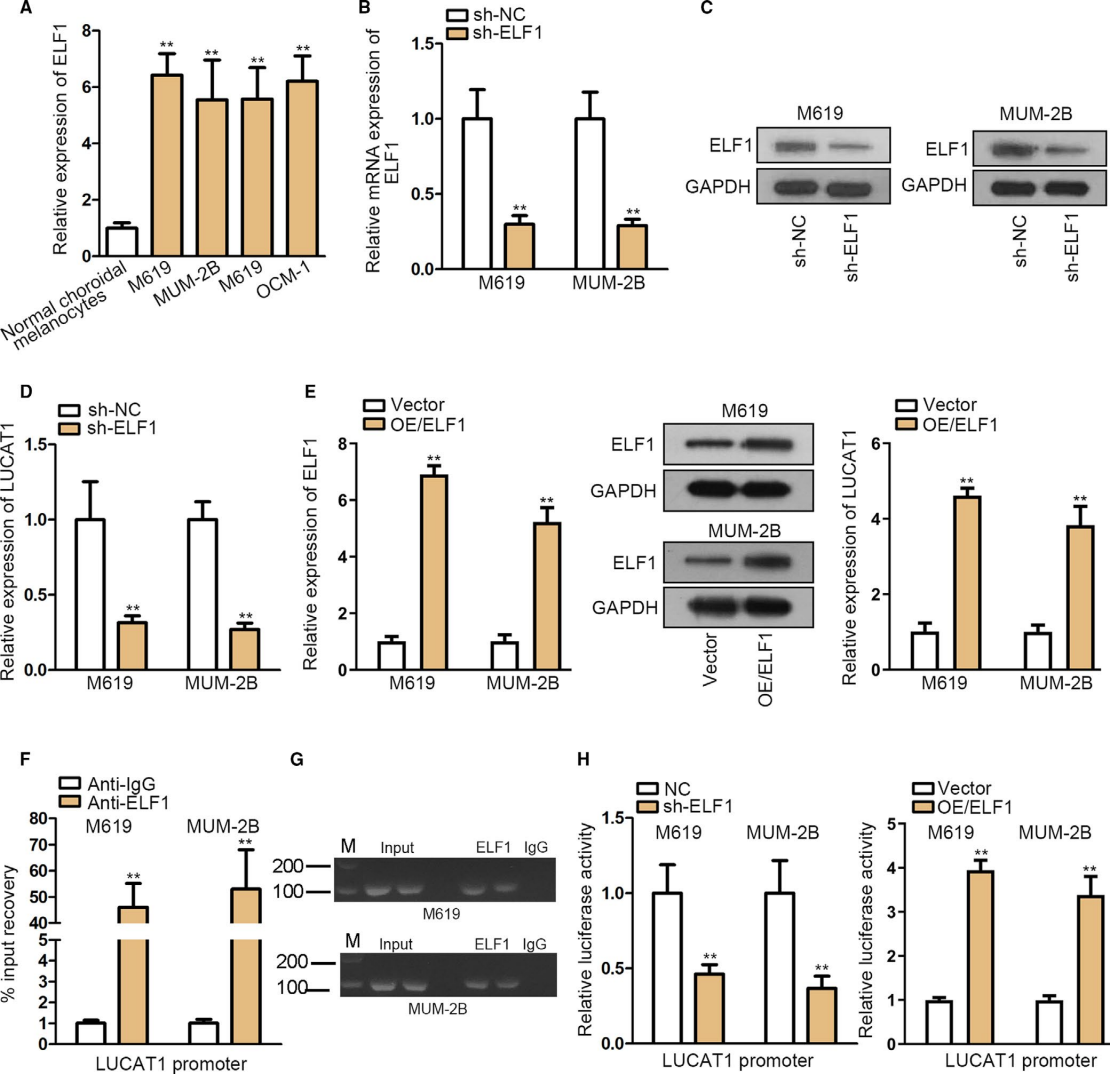
ELF1 is the transcription activator of LUCAT1. A, qRT‐PCR analysis of ELF1 relative expression in choroidal melanoma (CM) cells. B, Silencing efficacy of ELF1 in M619 and MUM‐2B cells. C, Protein level of ELF1 after silencing ELF1 mRNA. D, Reaction of LUCAT1 expression in response to ELF1 knockdown. E, The expression of ELF1 and LUCAT1 in CM cells with or without ELF1 overexpression was analyzed via qRT‐PCR or Western blot as needed. F, ChIP assay to detect the PCR product of ELF1 antibody. G, Agarose gel electrophoresis to verify the PCR product of ELF1. H, The impact of ELF1 on LUCAT1 transcription was demonstrated by luciferase reporter assay. **P < .01

To find out how the expression of LUCAT1 was elevated, firstly, we made the conjecture that LUCAT1 was transcriptionally activated by the upper stream transcription factor. To verify our hypothesis, we searched the possible transcription factors of LUCAT1 (Figure S1) from UCSC database (http://genome.ucsc.edu/). Interestingly, among these transcription factors, ELF1 was previously reported as a transcription activator of MEIS1.^11^ On this basis, we selected ELF1 for further study. In the meantime, we found the expression level of ELF1 was enhanced in CM cells (Figure 2A). Interestingly, along with decreased mRNA and protein levels of ELF1 caused by ELF1 knockdown (Figure 2B,C), we detected significant reduction in the expression of LUCAT1 (Figure 2D). In contrast, LUCAT1 expression was dramatically enhanced under ELF1 overexpression (Figure 2E). The subsequent chromatin immunoprecipitation assay and agarose gel electrophoresis assay proved the binding between LUCAT1 promoter and ELF1 (Figure 2F,G). At last, we conducted luciferase reporter assay and observed a prominent decrease after ELF1 silence, whereas a significant augment in response to ELF1 upregulation (Figure 2H), suggesting ELF1 transactivated LUCAT1 in CM.

### 3.3 LUCAT1 modulates miR‐514a/b‐3p expression by serving as a sponge

**Figure 3:**
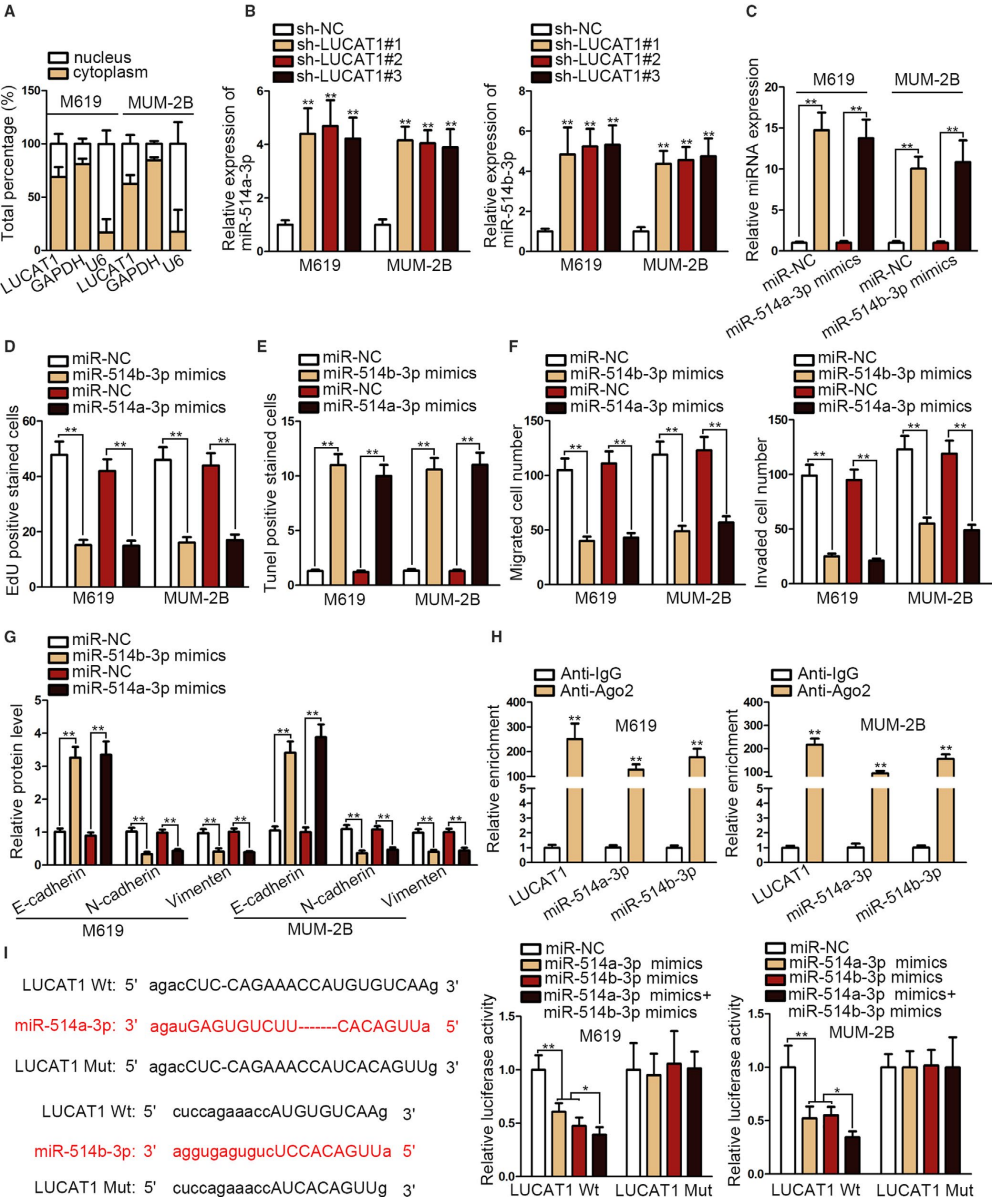
LUCAT1 modulates miR‐514a/b‐3p expression by serving as a sponge. A, RNA analysis of the nucleus and cytoplasm fraction to detect LUCAT1 location. B, MiR‐514a/b‐3p reaction in response to knockdown of LUCAT1. C, MiR‐514a/b‐3p expression after transfected miR‐514a/b‐3p mimics into cells. D and E, CCK‐8, EdU, and TUNEL assays to observe cell viability, proliferation, and apoptosis after overexpressing miR‐514a/b‐3p. F, Migrated and invaded cell number after overexpression of miR‐514a/b‐3p. G, Western blot assay presented EMT process after overexpressing miR‐514a/b‐3p. H, RNA immunoprecipitation assay verified the coexistence of indicated molecules in RNA‐induced silencing complex. I, Luciferase reporter assay confirmed the reaction between miR‐514a/b‐3p and LUCAT1. *P < .05; **P < .01

RNA analysis in nuclear and cytoplasmic fraction demonstrated LUCAT1 was predominantly located in the cytoplasm (Figure 3A). Through screening from StarBase (http://starbase.sysu.edu.cn/), we selected 17 miRNA candidates that might share binding sites with LUCAT1. Among these candidates, miR‐514a‐3p and miR‐514b‐3p were validated to be downregulated while others seemed to be unchanged in CM cells (Figure S2A). Besides, both miR‐514a‐3p and miR‐514b‐3p presented an increase in their relative expression after silencing LUCAT1 (Figure 3B). Thereafter, we overexpressed the miR‐514a/b‐3p to investigate their function in CM (Figure 3C). Results demonstrated overexpression of miR‐514a/b‐3p inhibited cell proliferation (Figure 3D). T**UNEL** assay proved miR‐514a/b‐3p upregulation increased apoptotic cell number (Figure 3E). Also, cell motility and EMT process were both obstructed after transfection of miR‐514a/b‐3p mimics into CM cells (Figure 3F,G). Intriguingly, RIP assay confirmed that LUCAT1 and miR‐514a/b‐3p were coexisted in RNA‐induced silencing complexes (RISCs) (Figure 3H). Through luciferase reporter assay, we observed that the luciferase activity of LUCAT1 wild‐type (LUCAT1 Wt) lowered in response to miR‐514a‐3p mimics or miR‐514b‐3p mimics and further decreased under cotransfection of miR‐514a‐3p and miR‐514b‐3p mimics. On the contrary, the luciferase activity of LUCAT1 mutant‐type (LUCAT1 Mut) showed no significant change under diverse conditions (Figure 3I).

### 3.4 RBX1 is the downstream target gene of miR‐514a/b‐3p

**Figure 4:**
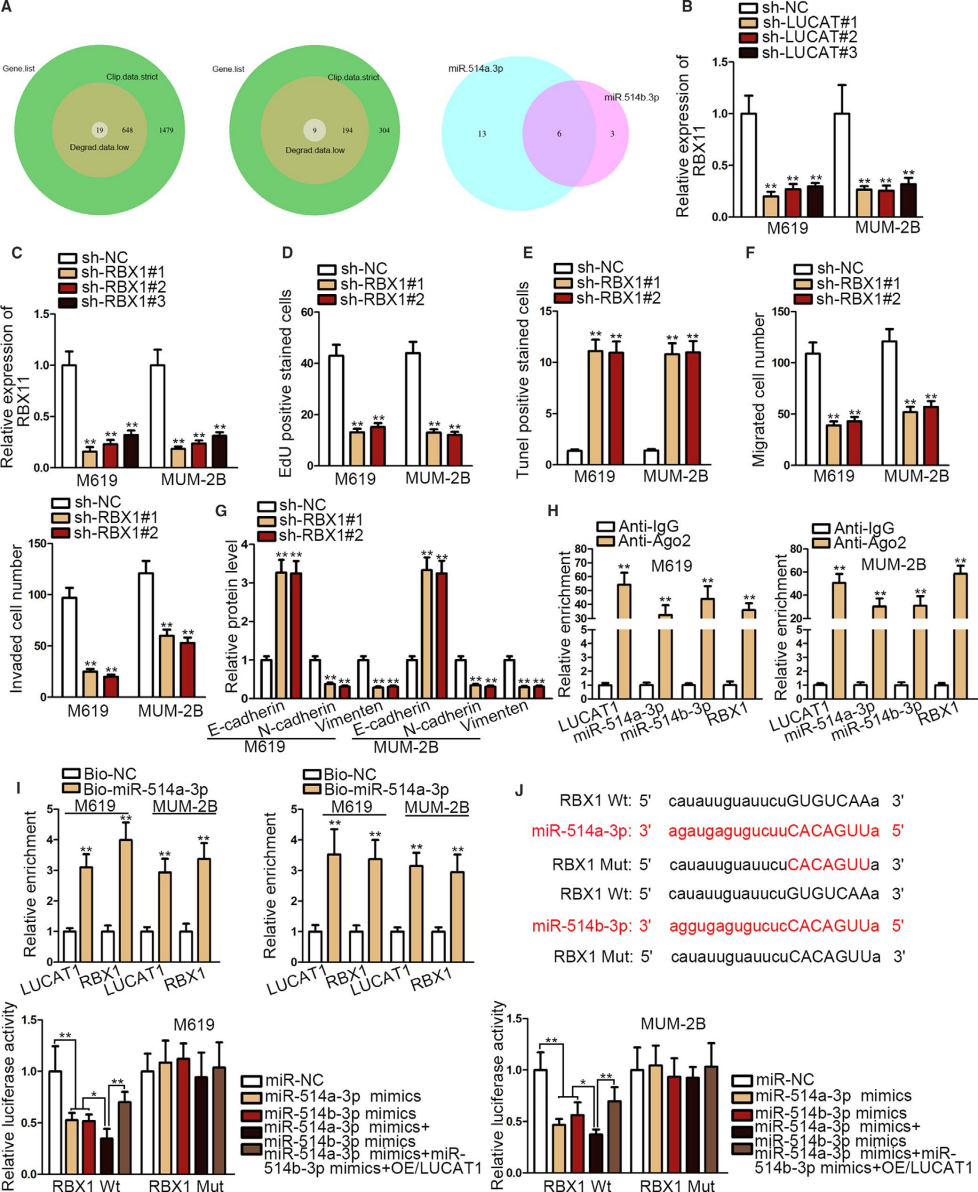
RBX1 is the downstream target gene of miR‐514a/b‐3. A, MRNA candidates screened from StarBase. B, RBX1 expression was significantly decreased after knockdown of LUCAT1. C, RBX1 knockdown efficiency examination. D‐G, Change in cell proliferation, apoptosis, motility, and EMT process after silencing RBX1. H, RNA immunoprecipitation assay proved the coexistence of the indicated molecules. I, Pull‐down assay suggested that the expression of LUCAT1 and RBX1 could be detected from biotinylated miR‐514a‐3p and miR‐514b‐3p. J, The interaction among the indicated molecules was verified by luciferase reporter assay. *P < .05; **P < .01

By screening from StarBase database, we selected 19 mRNAs that might bind to miR‐514a‐3p. And the screening conditions were set as clip data: strict, degradome data: low. Nine mRNAs were screened out since they might share binding sites with miR‐514b‐3p according to the following conditions: clip data: strict, degradome data: low. The Venn plotting presented that there were six targets shared between miR‐514a‐3p and miR‐514b‐3p (Figure 4A). To narrow down the mRNA candidates, we detected the relative expression of the six mRNAs in CM cells, uncovering that only RBX1 expression was upregulated in CM cells (Figure S2B). We also found a downregulation in RBX1 expression after knockdown of LUCAT1 (Figure 4B). Moreover, we silenced RBX1 in CM cells (Figure 4C) and then performed experiments to explore changes in CM cells after loss of RBX1function. EdU and T**UNEL** demonstrated, respectively, that silencing RBX1 attenuated cell proliferation and accelerated apoptosis (Figure 4D,E). Cell migration and invasion were also attenuated after knockdown of RBX1 (Figure 4F). Additionally, according to the results of western blot, EMT process was blocked after silencing RBX1 (Figure 4G). Therefore, we can conclude RBX1 functions as an oncogene in CM. Furthermore, RIP assay elucidated the co‐enrichment of RBX1, LUCAT1, miR‐514a‐3p, and miR‐514b‐3p in RISCs (Figure 4H). Pull‐down assay verified that both miR‐514a‐3p and miR‐514b‐3p could pull down RBX1 and LUCAT1 in the meantime (Figure 4I). Finally, the luciferase reporter assays indicated that the luciferase activity of RBX1 Wt declined after the upregulation of either miR‐514a‐3p or miR‐514b‐3p and further reduced in the context of miR‐514a/b‐3p co‐enhancement. However, such reduction could be attenuated after enforced expression of LUCAT1 (Figure 4J). The result indicates the interaction among the four molecules.

### 3.5 Overexpressed RBX1 restored the function loss due to silencing LUCAT1

**Figure 5:**
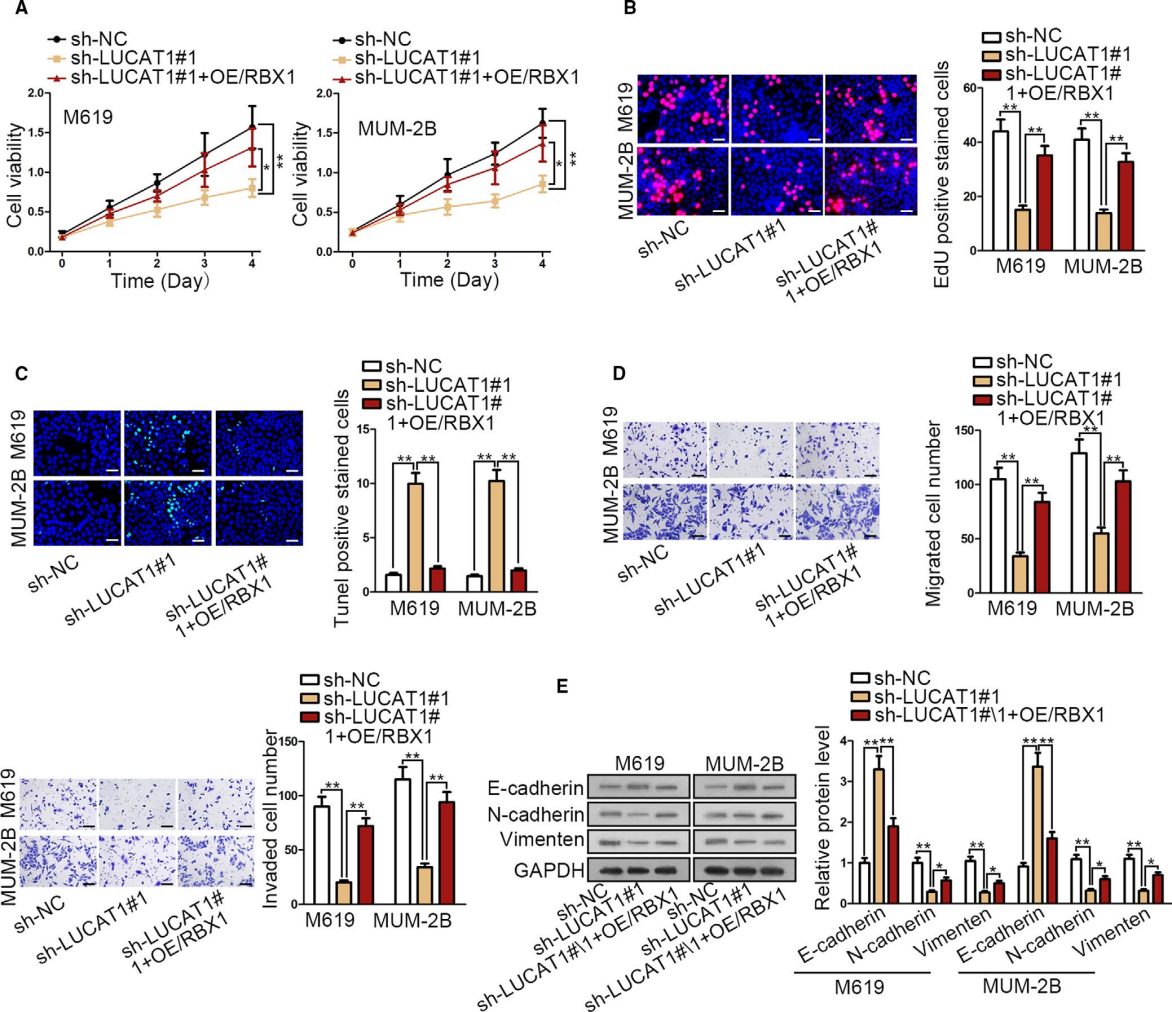
Overexpressed RBX1 restored the function loss due to silencing LUCAT1. A and B, CCK‐8 assay and EdU assay (scale bar = 200 μm) proved overexpressed RBX1 could save the decrease in cell viability and proliferation caused by silencing LUCAT1. C, TUNEL assay demonstrated that apoptotic cell number increased after LUCAT1 knockdown and recovered after overexpressing RBX1 (scale bar = 200 μm). D, Cell migration and invasion with the treatment of overexpressed RBX1 by transwell assays (scale bar = 200 μm). E, Western blot assay detected EMT process variation after choroidal melanoma cells transfected with overexpressed RBX1. *P < .05; **P < .01

At length, we planned to verify whether above unveiled LUCAT1/RBX1 pathway functioned in CM. Thus, rescue experiments were conducted subsequently. As anticipated, we observed a normalized effect in LUCAT1 inhibition‐affected cell viability, proliferation ability, and apoptosis in CM cells after being transfected with pcDNA3.1/RBX1 (OE/RBX1) (Figure 5A‐C). Transwell assays demonstrated that overexpressed RBX1 recovered the suppressed cell migration and invasion in LUCAT1‐silenced CM cells (Figure 5D). Also, according to Western blot result, EMT process blocked by silenced LUCAT1 was restored in face of RBX1 overexpression (Figure 5E). Taken that, we can conclude that LUCAT1 promotes CM progression by targeting miR‐514a/b‐3p/RBX1 axis.

## 4 DISCUSSION

Choroidal melanoma is an intraocular tumor with rare incidence of occurrence but relatively high death rate up to 50% among patients.^2^ LUCAT1 has been commonly recognized as an oncogene in various cancers including non‐small lung cancer, ovarian cancer, clear cell renal cancer, breast cancer, oral squamous cell carcinoma, etc.^12^, ^13^, ^14^, ^15^, ^16^ However, the expression of LUCAT1 and its functions in CM have not been explored yet. Here, we examined the upregulated expression of LUCAT1 in CM cells for the first time. The further loss‐of‐function experiments confirmed LUCAT1 as a tumor promoter in CM.

To find out whether there is an upstream regulator responsible for LUCAT1 upregulation, we used UCSC database to find possible transcription factor for LUCAT1 (Figure 2A). Among these transcription factors, ELF1 was recognized as a contributor to malignancies^17^, ^18^, ^19^ and it was also reported as a transcription activator of MEIS1^11^ but an inhibitor of erbB2 promoter activity.^20^ To investigate whether and how ELF1 modulates LUCAT1 expression, we first examined the expression of ELF1 in CM cells, finding that the expression of ELF1 is upregulated significantly, similar to ELF1 overexpression in endometrial carcinoma.^21^ We also observed that ELF1 acted as a transcriptional activator of LUCAT1 in CM.

Recently, cytoplasmic lncRNAs have been increasingly suggested to regulate disease development via acting as a ceRNA of protein‐coding genes through sponging certain miRNAs.^22^, ^23^ Meanwhile, many reports revealed that LUCAT1 could serve as a ceRNA to exert its facilitating role in various cancers. For example, LUCAT1 activated by STAT3 modulates hepatoblastoma by sponging miR‐301b and regulating STAT3 expression.^24^ LUCAT1 affects chemoresistance in osteosarcoma by targeting miR‐200c/ABCB1 axis.^25^ In our study, we found LUCAT1 mainly located in the cytoplasm and therefore conjectured LUCAT1 as a ceRNA to control the progression of CM. We further searched possible downstream miRNAs by StarBase. Subsequently, we focused on miR‐514a‐3p and miR‐514b‐3p derived from miR‐514 which seemed to play an antitumor role in cancer.^26^ Besides, miR‐514a‐3p was suggested as a tumor suppressor previously,^27^, ^28^ so was miR‐514b‐3p.^29^ Herein, we discovered that miR‐514a‐3p and miR‐514b‐3p were downregulated in CM cells and overexpressed miR‐514a/b‐3p inhibited cell proliferation and metastasis and EMT process. Further, we confirmed that miR‐514a/b‐3p interacted with LUCAT1 in RISCs in CM cells.

Six mRNAs might share binding sites with LUCAT1 were screened out using online bioinformatics analysis tool StarBase. Finally, RBX1 was screened out due to elevated expression in CM cells. Moreover, RBX1 was proved to promote CM cell proliferation and metastasis, consistent with several evidences which revealed RBX1 as an unfavorable factor in non‐small lung cancer, gastric cancer, etc.^30^, ^31^ More importantly, rescue assays further verified that RBX1 was the mediator for LUCAT1 to facilitate CM development.

In conclusion, ELF1 promotes transcription of LUCAT1 and the elevated expression of LUCAT1 accelerates CM progression by sponging miR‐514a/b‐3p and releasing LUCAT1.

## CONFLICT OF INTEREST

All authors declare that they have no competing interests.

## Data Availability

Research data and material are not shared.
