# Supplementary figures and images for: ELF1‐mediated LUCAT1 promotes choroidal melanoma by modulating RBX1 expression
Source: Cancer Med. 2020 Jan 22;9(6):2160–70. doi: 10.1002/cam4.2859 (PMC7064025; doi:10.1002/cam4.2859)

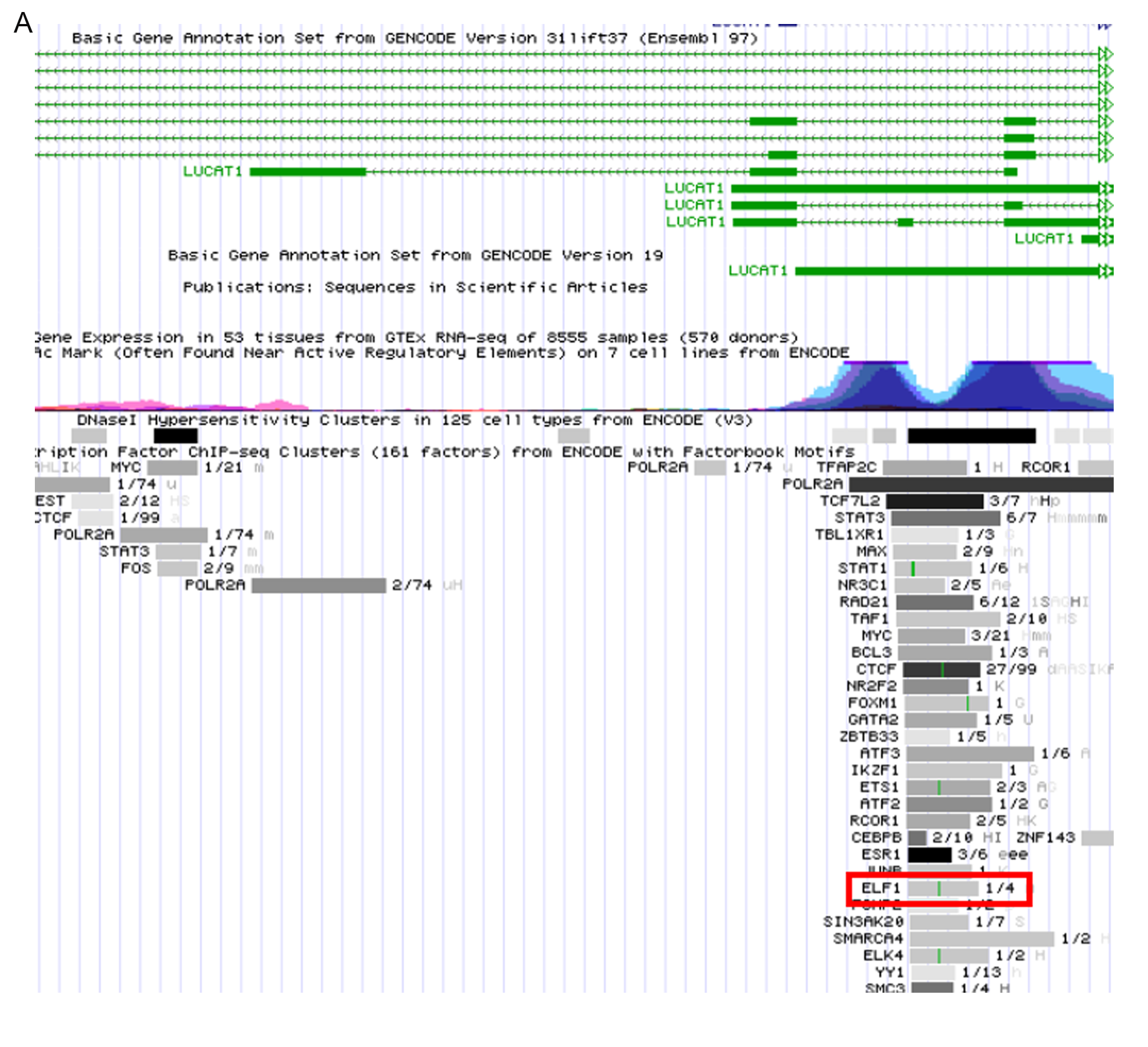

Supplement: Supplementary file 1 [file CAM4-9-2160-s001.tif]

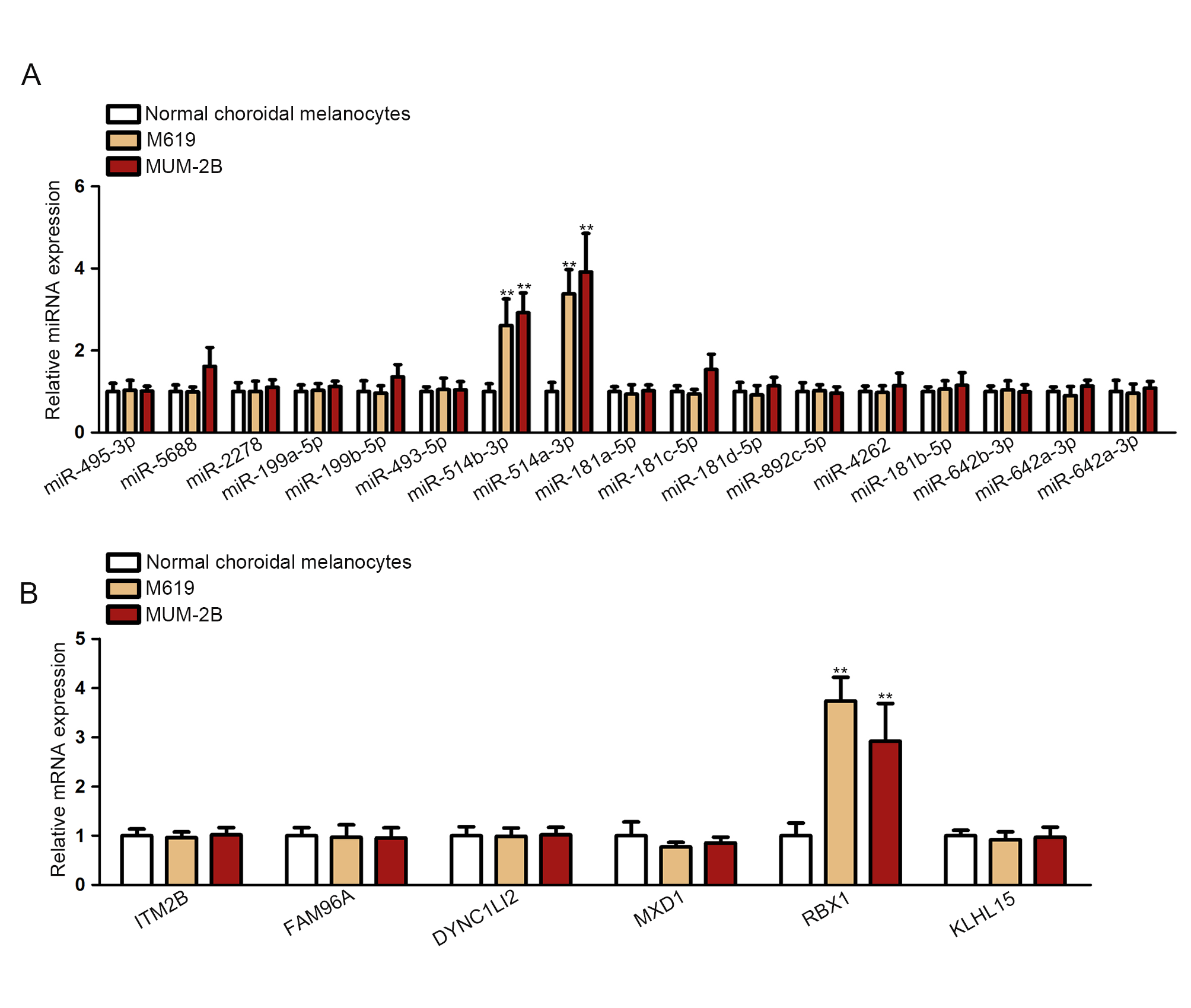

Supplement: Supplementary file 2 [file CAM4-9-2160-s002.tif]
